# *Equus* in Online Mendelian Inheritance in Animals (OMIA)

**DOI:** 10.3390/ani14142069

**Published:** 2024-07-15

**Authors:** Imke Tammen, Ernest Bailey, Marius Mather, Frank W. Nicholas

**Affiliations:** 1 Sydney School of Veterinary Science, Faculty of Science, The University of Sydney, Sydney, NSW 2006, Australia; frank.nicholas@sydney.edu.au; 2 Gluck Equine Research Center, University of Kentucky, Lexington, KY 40546, USA; ebailey@email.uky.edu; 3 Sydney Informatics Hub, The University of Sydney, Sydney, NSW 2006, Australia

**Keywords:** mendelian, trait, disease, genetic, variant, ontology

## Abstract

**Simple Summary:**

Online Mendelian Inheritance in Animals (OMIA) is a freely available online resource for information on inherited traits/diseases (called phenes) in animals, including *Equus caballus* (horse) and eight other *Equus* subgroups. Maintaining up-to-date information in OMIA is a major challenge, especially in relation to variants (mutations), as reference genomes continue to evolve. The site is curated by faculty at the University of Sydney based on publications of peer-reviewed research. Curation has been aided by contributions from faculty and students at other institutions. Recently, OMIA has introduced computerized lists of standardized names and synonyms (called ontologies) for breeds of horses and other animal species and for phene categories. These ontologies facilitate increased connectivity between OMIA and other online resources. OMIA is and will continue to be a major reference resource for Mendelian phenes in the genus *Equus*.

**Abstract:**

Online Mendelian Inheritance in Animals (OMIA is a freely available information resource, which includes information for *Equus* inherited traits/diseases (collectively called phenes). The database focuses on Mendelian traits and their likely causal variants (mutations). Some of these Mendelian traits are favored by humans, e.g., coat color, while others are diseases. Additions to OMIA are based on publications of peer-reviewed research. Maintaining up-to-date information in OMIA is a challenge, owing to the multiplicity of species, the increase in the number of relevant publications, and as reference genomes and methods of citation continue to evolve. This challenge has been successfully aided by contributions from scientists from around the world. In some cases, those scientists are faculty members who charge their students with curation as an educational activity. Recently, OMIA has introduced computerized lists of standardized names and synonyms (called ontologies) for breeds of *Equus* and other animals and for phene categories. These ontologies facilitate increased connectivity between OMIA and other online resources. OMIA is and will continue to be a major reference resource for Mendelian phenes in the genus *Equus*.

## 1. Introduction

OMIA (https://omia.org, accessed on 13 June 2024) [1] is a freely available, curated, online knowledgebase which provides users with up-to-date summary information on the known harmful and beneficial variants in animals, together with background information on known inherited disorders and beneficial traits. This resource grew out of a mainframe database created in 1980 by Frank Nicholas at the University of Sydney and is based on information extracted from research reports published in peer-reviewed scientific journals. The online version was launched in 1995. The site is currently curated by Imke Tammen, and software engineering support is provided by Marius Mather. OMIA is modeled on and reciprocally hyper-linked to Online Mendelian Inheritance in Man (OMIM, https://omim.org, accessed on 13 June 2024) [2] and provides further links to PubMed and Gene records at the National Center for Biotechnology Information (NCBI) [3] and the European Bioinformatics Institute (EBI)’s Ensembl [4]. Recent links have been created to the Mondo Disease Ontology (Mondo, https://mondo.monarchinitiative.org, accessed on 13 June 2024) [5] and the newly developed Vertebrate Breed Ontology (VBO, https://www.ebi.ac.uk/ols4/ontologies/vbo, accessed on 13 June 2024) [6].

OMIA focuses on traits and diseases (‘phenes’) with confirmed or suspected Mendelian modes of inheritance, i.e., they are inherited in a manner consistent with a variant in just one gene. In addition, several phenes with unknown or complex modes of inheritance and phenes caused by somatic mutations, genetic modifications or genome editing are also included. While most OMIA entries are for the major domesticated animal species, more than 500 (mainly vertebrate) animal species have entries in OMIA. Information about humans and model organisms such as mice, rats, zebrafish and western clawed frogs is not included as they have dedicated species-specific resources.

In this paper, we provide an overview of OMIA data relevant to the taxonomic group *Equus*, discuss the relevance to horse breeding and genetics, and report how students have contributed to OMIA curation of these entries.

## 2. *Equus* in OMIA

### 2.1. Equus Species in OMIA

OMIA lists information for nine taxonomic subgroups in the genus *Equus*: *Equus caballus* (horse), *Equus asinus* (ass/donkey), *Equus asinus* × *Equus caballus* (mule/hinny), *Equus grevyi* (Grévy’s zebra), *Equus hemionus* (onager), *Equus kiang* (kiang), *Equus przewalskii* (Przewalski’s horse), *Equus quagga* (plains zebra) and *Equus zebra* (mountain zebra). Most of the information relates to *Equus caballus*.

### 2.2. Equus Traits and Diseases (Phenes) in OMIA

OMIA lists information for 74 Mendelian (single-locus) *Equus* phene species, which are summarized in Table 1. Also listed in this table are the number of Mendelian phenes with at least one known likely causal variant and the total number of known likely causal variants. The last column of Table 1 presents the total number of other *Equus* phenes included in OMIA, namely those (as mentioned above) with unknown or complex modes of inheritance or those which are caused by somatic mutations, genetic modifications or genome editing. For detailed information on all *Equus* phenes see Supplementary Table S1.

An important new feature of OMIA is that each phene is allocated to one set of standardized categories, mostly gleaned from the major biological system headers of the Mammalian Phenotype Ontology (MPO) [7]. The ‘Browse’ tab in OMIA provides a table that allows for the identification of phenes within each category for different species. This feature is particularly useful for veterinarians when they suspect an inherited disease, are presented with clinical signs in a specific body system and want to compile a list of differential diagnoses. Table 2 shows the number of *Equus caballus* phenes for each category.

OMIA presents variant information in downloadable tables that may include information about breed, variant phenotype, gene, allele, type of variant (e.g., missense, nonsense, deletion, insertion, etc.), source of genetic variant (e.g., naturally occurring versus genetic modification/genome editing), variant coordinates, European Variation Archive (EVA) [8] identifier, as well as the year and PubMed identifier for the first report of the variant. An acknowledgement field and a verbal description field allow curators to provide additional commentary. This can be used to create awareness about conflicting evidence in the published literature regarding the causality of a variant or identify limitations in the evidentiary support of causality. OMIA aims to present variant nomenclature information based on recommendations of the Human Genome Variation Society (HGVS) [9]. HGVS nomenclature is the internationally recognized standard for the reporting of genetic variants in the medical context as it allows for unambiguous naming of all types of variants, including series of variants on the same chromosome (haplotypes), large structural variants and complex variants. In contrast to some other variant call formats, the variant description is unambiguous, without the need to link to additional metadata, and includes information at the DNA level (g. or m.) as well as coding (c.) and protein (p.) nomenclature to facilitate functional interpretations [9]. As many variants were reported before reference sequences were available, and as variant information is frequently not published following HGVS recommendations, standardizing variant information in OMIA has been a major curation task and required geneticists with species expertise to review original publications and often conduct bioinformatic analyses. In 2021, 50 OMIA-listed horse variants were submitted to the European Variation Archive (EVA) [8] to be assigned rs identifiers. In accordance with HGVS recommendations, OMIA is currently implementing the addition of sequence identifiers for genomic, transcript and protein coordinates for each variant. This process has now been completed for horse and ass variants (Supplementary Table S2). OMIA variant IDs are provided for each variant as a simple, unique and unchanging identifier to reduce confusion due to the changing of variant coordinates because of releases of new reference genomes. Where possible, regular automated tests are conducted to check that listed variants correspond to their reference sequences. Table 3 summarizes the key information on horse and ass variants.

Other relevant information about the phenes, genetics (e.g., details of the mode of inheritance, genetic testing, mapping information as well as information about non-causal variants in the DNA that are associated with a phenotype) as well as disease-related information can be added by curators in open text fields.

One of the little-noticed features of OMIA is the lists of papers under the home-page tab labeled Landmarks/Reviews/Resources. The first two categories are self-explanatory: landmark papers have made notable contributions to our knowledge, and reviews summarize the knowledge relevant to OMIA. The third category (called resources) comprises papers describing linkage maps (in the pre-genomic era), genome assemblies and other genome-related papers. In each category, the papers are listed separately for each species. Since these papers are arranged in reverse chronological order, a quick scan of the papers from bottom to top provides a historical timeline of the material covered by each category of papers. Table 4 provides a summary of the number of review and resource papers for *Equus* species.

### 2.3. Discussion

OMIA contains more entries for *Equus caballus* than for other *Equus* species, reflecting the greater amount of research devoted to the domestic horse. Furthermore, many of the entries (70 out of 107 likely causal variants) relate to pigmentation traits, reflecting the ease in phenotyping coat color as well as the interest in breeding horses with specific coat colors. It is also possible that OMIA curators have missed some important non-horse publications. If anyone spots such an omission, please contact omia.admin@sydney.edu.au.

OMIA aims to provide a summary of phene and variant information that is readily (and freely) accessible for practicing veterinarians, researchers, breeders, owners and genotyping service providers. In doing so, it aims to provide this information using standardized nomenclature for phenes, breeds and variants. To this end, in recent years, OMIA has devoted considerable time and effort to standardizing variant information and incorporating ontologies for phenes and breeds. At OMIA’s instigation, the Monarch Initiative is now leading a collaborative team in the creation and continual development of the Vertebrate Breed Ontology (VBO) [6]. First launched on 6 May 2022, the VBO provides free access to a comprehensive computerized list of standardized breed names, breed name IDs and breed name synonyms for all species that have breeds. This enables the inter-connection of relevant information on breeds from internet resources around the world. That same collaborative team is also incorporating animal phenes from OMIA into existing global phene ontologies such as Mondo, which in turn will provide a means of not only interlinking information on animal traits and diseases around the globe, but also increasing the reciprocal sharing of trait and disease information on humans and non-human animals.

Even with the new AI literature searching tool that was launched on 13 February 2023, OMIA curation is a time-consuming task. Inevitably, there is a continual need for checking, gap filling, updating and generally enhancing OMIA entries. Fortunately, this enhancement can be achieved in an educational context, as described in the next section.

## 3. OMIA Curation as an Authentic Learning Activity

From OMIA’s earliest days, attempts have been made to devise OMIA enhancement projects that would be suitable for undergraduate or postgraduate students. A major limitation of this strategy has always been the time required to devise a particular assignment that will match the state of knowledge of the students; other limitations are the time required to assess student performance in such assignments, and the time required to confirm or correct the students’ contributions before incorporating them into OMIA.

Since 2020, one of us (I. Tammen) has successfully mentored individual animal science and DVM student research projects in reviewing and enhancing a specific subset of OMIA entries, e.g., [10]. She has also mentored DVM class exercises in reviewing and enhancing the clinical and pathology content of OMIA entries and has mentored PhD students in reviewing and enhancing entries that relate to their PhD research project, e.g., [11].

Commencing in 2017, one of us (E. Bailey) offered an annual course to graduate and undergraduate students involving the curation of OMIA at the University of Kentucky. In particular, the Kentucky students have updated earlier reports in light of more recent changes in rules for nomenclature and with the evolution of reference genomes. Based on this work, the curation of text fields was changed for 16 horse phenes and information for 25 variants was updated between 2019 and 2024 (Supplementary Table S3).

In all of these cases, students who have contributed enhancements to OMIA entries have been acknowledged by name in the relevant entries. These exercises have been mutually beneficial: students have gained a greater understanding and appreciation of the complexities in the practical application of genetics and of the fundamental need for clear and concise writing; OMIA has gained better entries.

## 4. Practical Use of Information in OMIA

OMIA reflects the published literature on equine genetic traits and variants, providing a concise summary of peer-reviewed publications related to Mendelian traits. Of the 74 traits with evidence of Mendelian inheritance in *Equus* species, 56 have had likely causal variants identified. For each of these, a list of all known likely causal variants is provided, summarizing the key information about each variant, including, in most cases, the breed(s) in which that particular variant has been documented. In most of these cases, a DNA test for the potentially relevant variants could be very useful in enabling mating to be arranged so as to avoid the occurrence of disease or to create desirable non-disease traits. Commercial tests are available for many of the variants listed in OMIA and researchers use the standardized information in the variant tables as the basis for population studies and bioinformatic variant filtering tools [12,13,14]. However, it is important to note that OMIA is a reflection of the published literature and not an evaluation nor endorsement of any tests. To that end, a recently formed Working Group of the International Society for Animal Genetics (ISAG) will evaluate and assess the quality of evidence for variants. As soon as the classifications become available, they will be included in OMIA variant tables. This will provide very useful practical guidance, not only to breeders and veterinarians, but also to DNA genotyping providers; only those variants classified as pathogenic or likely pathogenic should be used in DNA tests.

OMIA also provides a list of all known *Equus* Mendelian phenes for which, so far, likely causal variants have not been reported. At the time of writing, there are 18 such Mendelian phenes, 11 of which are disease traits. If any of these Mendelian diseases appear, it would be possible to substantially decrease the chance of the occurrence of future cases by simply applying the principles of Mendelian inheritance. To do this requires feeding all available pedigree and occurrence information into the segregation analysis package called GeneProb (https://bkinghor.une.edu.au/geneprob.htm, accessed on 9 July 2024) [15]. Many animal geneticists will be able to provide help with running this package. It is an exceedingly powerful tool for the practical application of Mendelian principles.

OMIA also lists information about non-Mendelian traits. The information is mostly restricted to listing key references. For some of these phenes, curator comments are added in OMIA’s open text fields. Information about multifactorial or quantitative traits in OMIA partially overlaps with data presented in the Animal QTL database (QTLdb) [16]. QTLdb is restricted to presenting information for only eight species including horses (HorseQTLdb: https://www.animalgenome.org/cgi-bin/QTLdb/EC/browse, accessed on 9 July 2024), but for these species provides association data about quantitative traits in much greater detail, and the data are curated into structured tables managed in a relational database environment.

## 5. Conclusions

Obviously, the numbers of particular OMIA entries mentioned in this paper are subject to change as knowledge increases. Indeed, one of the major advantages of a resource such as OMIA is that it aims to always be up-to-date. Given that almost all of the daily curation is carried out by just one person (I. Tammen), who already had a full-time job before taking on the role of OMIA curation, it is inevitable that sometimes there will be a delay before recently published information is included in OMIA. We encourage anyone who spots an omission or an error to send an email to omia.admin@sydney.edu.au. While OMIA and OMIA downloads are freely available, we request that any resources or publications that result from the use of OMIA data cite OMIA [1] and, when relevant, contact us to discuss co-authorship to help us to continue to maintain and update this resource.

## Figures and Tables

**Table 1:** *Equus* phenes (disease and non-disease traits) and variants in OMIA (https://omia.org, accessed on 13 June 2024).

Common Name	All Phenes	All Mendelian Phenes	Mendelian Phenes with at Least One Known Likely Causal Variant	All Known Likely Causal Variants	Other Phenes *
Horse	293	63	48	107	230
Ass/donkey	32	9	7	8	23
Mule/hinny	9	0	0	0	9
Przewalski’s horse	5	2	1	1	3
Onager	3	0	0	0	3
Mountain zebra	3	0	0	0	3
Plains zebra	4	0	0	0	4
Grévy’s zebra	4	0	0	0	4
Kiang	2	0	0	0	2
TOTAL	355	74	56	116	281

* Phenes with unknown or complex modes of inheritance or which are caused by somatic mutations, genetic modifications or genome editing.

**Table 2:** *Equus caballus* entries summarized by OMIA category. OMIA categories are based on Mammalian Phene Ontology [7] terminology (https://omia.org, accessed on 13 June 2024).

OMIA Category	Total Phenes	Phenes with Known Likely Causal Variants *	Likely Causal Variants per Category *
Skeleton phene (incl. short stature and teeth)	33	4	8
Pigmentation phene	24	18	70
Hematopoietic system phene	22	1	2
Nervous system phene	22	4	4
Reproductive system phene	21	2	6
Vision/eye phene	20	6	7
Integument (skin) phene	19	8	8
Limbs/fins/digit/tail phene	18	2	3
Cardiovascular system phene	16	0	0
Homeostasis/metabolism phene	14	2	2
Immune system phene	13	2	2
Endocrine/exocrine gland phene (incl. mammary gland)	12	1	1
Muscle phene	12	5	5
Digestive/alimentary phene	10	1	1
Growth/size/body region phene	12	2	5
Behavior/neurological phene	9	0	0
Hearing/vestibular/ear phene	8	2	13
Respiratory system phene	8	0	0
Neoplasm	7	1	1
Mortality/aging (incl. embryonic lethal)	6	2	36 **
Craniofacial phene	6	0	0
Renal/urinary system phene	6	0	0
Normal phene	4	1	1
Embryo phene	4	0	0
Chromosomal disorder	3	0	0
Adipose tissue phene	2	0	0
Lysosomal storage disease	1	0	0
Liver/biliary system phene	1	0	0
Cellular phene	0	0	0
Taste/olfaction phene	0	0	0

* Each phene is allocated to at least one OMIA category; the same variant can therefore be counted multiple times in this table. ** This includes 35 *KIT* variants for dominant white coat color (OMIA: 000209-9796). Some but not all of the *KIT* variants are thought to be homozygous embryonic lethal.

**Table 3:** Summary of variant information for horse and ass species in OMIA (https://omia.org, accessed on 13 June 2024).

	Horse	Donkey/Ass
Likely causal variants	107	8
Variant coordinates in HGVS [9] format	101 *	8
Breed in VBO [6] format listed **	105	4
Variants with EVA [8] rs ID	66	0

* Reference sequence coordinates are missing for some structural variants. ** Breed information is limited for some variants to information from the original publication. For others, breed information has been updated to include data from multiple publications, including recent population studies, e.g., [10].

**Table 4:** *Equus* review and genomic resource papers in OMIA (https://omia.org, accessed 13 June 2024).

Common Name	Review Papers	Genomic Resources and Maps
Horse	144	55
Ass/donkey	1	10
Mule/hinny	0	0
Przewalski’s horse	0	3
Onager	0	3
Mountain zebra	0	2
Plains zebra	0	2
Grévy’s zebra	0	2
Kiang	0	4
TOTALS	145	81

## Data Availability

The data presented in this study are available in Online Mendelian Inheritance in Animals at https://omia.org, https://doi.org/10.25910/2AMR-PV70.

## Supplementary Materials

The following supporting information can be downloaded at: https://www.mdpi.com/article/10.3390/ani14142069/s1, Table S1: Equus phenes in OMIA (accessed 13 June 2024); Table S2: *Equus* variants in OMIA (accessed 13 June 2024); Table S3: University of Kentucky student contributions to OMIA curation.
